# A Cluster of Risks: Correlates of Energy Drink Consumption with Smoking, Diet, and Burnout in the Polish Adult Population

**DOI:** 10.3390/nu17233747

**Published:** 2025-11-28

**Authors:** Adrianna Szalonka, Anna Zimny-Zając, Siddarth Agrawal, Grzegorz Mazur, Aleksandra Butrym

**Affiliations:** 1 Division of Innovation in Health Care, Department of Public Health, Faculty of Health Sciences, Wroclaw Medical University, 50-367 Wroclaw, Poland; 2 Ringier Axel Springer Poland, 02-672 Warsaw, Poland; 3 Faculty of Health Science, Institute of Public Health, Jagiellonian University Medical College, 31-008 Kraków, Poland; 4 Labplus R&D, 50-266 Wroclaw, Poland; 5 Department of Emergency Medical Services, Faculty of Nursing and Midwifery, Wroclaw Medical University, 50-367 Wroclaw, Poland; 6 Clinic of Hematology and Oncology, Wroclaw Medical University, Branch in Walbrzych, 58-300 Walbrzych, Poland; aleksandra.butrym@gmail.com

**Keywords:** energy drinks, caffeine, public health, risk factors, mental health, sleep disorders, cross-sectional study

## Abstract

**Background**: We examined the prevalence and correlates of energy drink (ED) consumption in Polish adults using an archival, nationally sourced dataset. **Methods**: Cross-sectional analysis of 120,000 adults from the archival 2024 National Health Test of Poles (computer-assisted web interview). ED consumption was assessed by frequency and dichotomized for regression (ever vs. never). Multivariable logistic regression estimated adjusted odds ratios (aOR) with 95% confidence intervals; an age cut-off was derived using ROC/Youden. Owing to the cross-sectional design, all estimates are interpreted as associations rather than causal effects. **Results**: In this national sample, 16.9% of adults reported ever consuming energy drinks, while regular (weekly or more frequent) consumption was rare (2.8%). After multivariable adjustment, the strongest independent correlates of ever consuming an energy drink were an age ≤53 years (aOR 3.80, 95% CI 3.61–4.01), male sex (aOR 3.17, 95% CI 3.03–3.32), frequent fast-food consumption (aOR 2.63, 95% CI 2.51–2.76), and being a current smoker (aOR 2.49, 95% CI 2.23–2.77). In contrast to the initial hypothesis, consumption was not found to be independently associated with education level. A strong, dose-dependent relationship was observed between consumption frequency and an increased prevalence of sleep disturbances, depression, and somatic complaints like headaches and chest pain. **Conclusions**: Energy drink consumption in Poland is concentrated within a high-risk demographic of young to middle-aged men and is deeply embedded within a cluster of adverse health behaviors. These findings underscore the need for comprehensive public health interventions that address the entire lifestyle pattern, rather than focusing solely on energy drink use.

## 1. Introduction

The consumption of energy drinks represents a significant and escalating public health issue globally. A recent meta-analysis covering over one million respondents estimated that more than half of the population (54.7%) has consumed an energy drink at some point, with over a fifth (21.6%) reporting use within the last week [1]. These products are defined as non-alcoholic beverages with high concentrations of caffeine (≥150 mg/L) and sugars, often fortified with other stimulants such as taurine, ginseng, and guarana [2]. While the primary pharmacological effect is attributed to caffeine’s role as an adenosine receptor antagonist, other additives may have a synergistic, though less documented, impact [3,4]. This growing market is accompanied by a complex regulatory landscape, where inconsistent labeling requirements hinder direct comparisons between studies and complicate clinical guidance [3,4]. The widespread use of these products, particularly among adolescents and young adults, is concerning given the mounting evidence of associated health risks [1].

The adverse health consequences of energy drink consumption are multifaceted. A primary concern relates to their impact on sleep, with meta-analyses indicating a significantly increased risk of insomnia and hyperarousal among consumers [5]. These findings are consistent with interventional studies on caffeine, which demonstrate a dose-dependent deterioration of key sleep parameters, including reduced total sleep time, lower efficiency, and prolonged sleep latency [6]. Secondly, significant cardiovascular effects have been documented, including acute increases in blood pressure and, in some studies, repolarization changes such as QTc prolongation [7,8]. Notably, some research suggests these effects are greater than those from an equivalent dose of caffeine alone, implicating other ingredients in the cardiovascular response [9]. Finally, consumption has been linked to increased risk-taking behaviors, particularly the practice of mixing energy drinks with alcohol (AmED). Consumers of AmED report higher rates of hyperarousal compared to those who drink alcohol alone [10], leading major health bodies and researchers to strongly discourage this combination [7,11,12].

Beyond these direct health effects, emerging evidence suggests that energy drink consumption is not an isolated behavior but part of a cluster of health risk factors. Prior studies have consistently identified associations with male gender and younger age groups, including among university students [13] and the general adult population in Poland [14]. Furthermore, research has highlighted the frequent co-use of energy drinks with other substances, including tobacco and cannabinoids [13]. However, much of this research relies on smaller or regional samples that lack the statistical power to disentangle these interconnected variables and identify independent correlates. Therefore, a significant gap remains in understanding the complete sociodemographic and behavioral profile of energy drink consumers within a large, nationwide population after controlling for key confounders. This is particularly salient in Poland, where legislation enacted on January 1, 2024, prohibited the sale of energy drinks to minors, bringing the issue to the forefront of public health discourse and underscoring the need for robust, population-level data [15].

Accordingly, this study leverages the comprehensive dataset from the “National Health Test of Poles 2024,” a survey of 120,000 participants, to address these critical gaps. The primary aims of this analysis were to conduct the following: (1) determine the prevalence and patterns of energy drink consumption in a large, nationwide sample of Polish adults; (2) identify the independent sociodemographic and behavioral correlates of consumption, with a specific focus on clustered risk factors; and (3) examine the associations between energy drink use and a range of adverse health and social consequences.

## 2. Methods

This study was a cross-sectional analysis of data from the 5th edition of the National Health Test of Poles (NHTP 2024), a nationwide, voluntary online survey administered via a Computer-Assisted Web Interview (CAWI). The final sample for this analysis comprised 120,000 adult respondents. The final analytic sample included all NHTP 2024 respondents aged ≥18 years with complete data on energy drink consumption and core covariates (sex, age, place of residence, education, smoking status, problem drinking, and fast-food consumption). Respondents with missing data for the primary exposure or key adjustment variables were excluded from regression analyses.

The primary outcome was the frequency of energy drink consumption, measured with the question: “How often do you drink energy drinks?”. For descriptive reporting, responses were grouped into four categories based on consumption frequency: never, once a month or less, once a week or less, and at least once a day. For descriptive purposes, ‘regular use’ was defined as consumption at least once per week (categories ‘once a week or less’ and ‘at least once a day’). For regression modeling, this variable was dichotomized to compare respondents who had ever consumed an energy drink (“ever” users) with those who had never consumed one (“never” users). All findings are interpreted as pertaining to consumption frequency irrespective of product composition, brand, or serving size.

A comprehensive set of covariates was analyzed. Sociodemographic characteristics included sex, age, place of residence, and education level. Health behaviors were assessed through questions on tobacco smoking status, alcohol consumption patterns, from which a binary variable for ‘problem drinker’ was derived based on frequency of binge drinking, and the frequency of fast-food intake. Health status was evaluated based on the self-reported presence of chronic diseases, such as hypertension, heart disease, diabetes, COPD, cancer, and joint diseases. Additionally, the analysis included the frequency of various symptoms and complaints, such as insomnia, daytime sleepiness, headaches, chest pain, heartburn, limb numbness, urinary incontinence, constipation, memory problems, and work-related exhaustion. Somatic complaints, sleep problems, and depressive symptoms were assessed using single self-report items from the NHTP 2024 questionnaire, with responses recorded on a three-level frequency scale (never, occasionally, at least several times per month). These items are not equivalent to formal clinical diagnoses but serve as standardized screening-type indicators across the full sample.

All statistical analyses were performed using STATISTICA 13.3 (TIBCO) and R 4.4.3, with the significance level for all two-sided tests set at α = 0.05. The distribution of continuous variables was assessed for normality using the Kolmogorov–Smirnov test. Descriptive statistics were presented as means and standard deviations (SD) for normally distributed variables or as medians and interquartile ranges [Q1; Q3] for non-normally distributed variables. Categorical variables were summarized as counts (*n*) and percentages (%). Initial associations between these variables and the ordinal scale of energy drink consumption were examined using Pearson’s χ^2^ test for categorical variables, the Mann–Whitney U test for comparisons between two groups, and the Kruskal–Wallis test for comparisons across more than two groups.

To identify factors independently associated with energy drink consumption, a multivariable logistic regression model was developed using the dichotomous “ever” versus “never” outcome. Initially, univariate logistic regression was performed for each potential explanatory variable. Variables with a *p*-value < 0.25 in the univariate analysis were considered candidates for inclusion in the final multivariable model. The model’s results are presented as odds ratios (OR) with 95% confidence intervals (CI). As part of the modeling process, the continuous variable of age was dichotomized to improve interpretability. The optimal age cut-off point was determined using Receiver Operating Characteristic (ROC) curve analysis, with the threshold selected to maximize the Youden index. The area under the curve (AUC), sensitivity, specificity, and positive likelihood ratio (LR+) were calculated to evaluate the discriminatory performance of this cut-off. All regression coefficients are interpreted as non-causal associations, given the cross-sectional study design and the potential for reverse causation and residual confounding. In other words, the models capture patterns of co-occurrence between energy drink consumption and participants’ characteristics rather than predictive or causal effects.

A key consideration for interpreting all results was the study’s substantial sample size. With N = 120,000, the statistical power of the tests approached 1.00, meaning that even minor, clinically irrelevant differences could achieve statistical significance. Therefore, the interpretation of the findings prioritized the magnitude of the effect sizes, such as odds ratios, and applied a threshold for practical significance in addition to *p*-values.

## 3. Results

### 3.1. Characteristics of the Study Population

The analysis included 120,000 adult respondents. The mean age was 57.7 ± 14.4 years, with a median of 60 years (range 18–99), and women constituted 62.7% of the sample. The population was educationally diverse, with 39.3% holding a Master’s degree, 29.4% having completed secondary education, and 2.2% primary education. A majority of participants (67.2%) reported having at least one chronic disease. The most frequently reported complaints (occurring at least several times per month) were back pain (38.9%), insomnia (27.2%), and daytime sleepiness (26.2%).

### 3.2. Prevalence of Energy Drink Consumption

Among the 120,000 participants, 16.9% (*n* = 20,250) reported having ever consumed an energy drink. The distribution of consumption frequency was highly skewed towards non-use, with 83.1% (*n* = 99,750) reporting they never consume these products. Sporadic consumption (once a month or less) was reported by 11.8% of the sample. Regular, frequent use was rare: 4.5% of respondents reported consuming energy drinks at least once a week, and only 0.6% reported daily consumption (Table 1).

### 3.3. Sociodemographic and Behavioral Correlates

**Figure 1:**
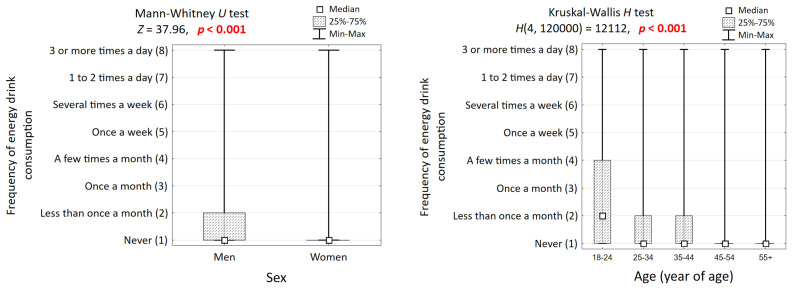
Comparison of the frequency of drinking energy drinks in groups of respondents differing in gender and age, and the results of significance tests.

Energy drink consumption was significantly associated with all analyzed sociodemographic and behavioral characteristics (all *p* < 0.001) (Table 2). A distinct age gradient was observed: the proportion of individuals aged 18–24 rose from 0.9% in the non-consumer group to 13.1% in the daily consumer group, while the proportion of those aged 55+ decreased from 66.6% to 16.9% across the same groups. Consumption was significantly more frequent among men, who constituted only 33.6% of non-consumers but over half of all consumer groups (Figure 1). Higher consumption frequency was also associated with residence in the largest cities (≥500,000 inhabitants), where the prevalence increased from 14.1% among non-consumers to 26.3% among daily consumers. The relationship with education was non-linear; while sporadic consumption was most common among those with higher education, daily consumption was most prevalent among those with a basic education. Similarly, consumption was highest at the extremes of the BMI distribution, among those who were underweight or obese.

**Figure 2:**
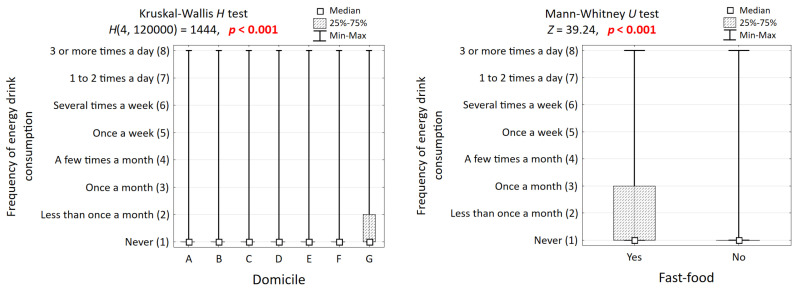
Comparison of the frequency of drinking energy drinks in groups of respondents differing in place of residence and frequent consumption of fast food, and the results of significance tests.

Strong, dose-dependent relationships were evident for risk behaviors. The prevalence of current smoking showed a stark gradient, rising from 1.4% among non-consumers to 16.2% among daily consumers. A similar pattern was observed for problem drinking, which increased from 5.8% in non-consumers to 16.0% in the daily use group. Frequent fast-food consumption was also significantly more common among energy drink users (Figure 2).

### 3.4. Association with Health Status and Symptoms

The prevalence of chronic diseases and self-reported symptoms varied significantly with the frequency of energy drink consumption (Table 3). Monotonic trends were observed for several conditions: the prevalence of hypertension systematically decreased from 46.4% in non-consumers to 26.9% in daily consumers, while the prevalence of depression increased from 17.1% to 36.5% across the same groups.

The frequency of self-reported symptoms showed a consistent dose–response relationship with energy drink use (Table 4 and Table 5). The strongest correlations were observed for work-related exhaustion, where the proportion of those experiencing it frequently rose from 24.2% in non-consumers to 54.4% in daily consumers (*p* < 0.001), and daytime sleepiness, which increased from 24.6% to 46.8% across the same groups (*p* < 0.001).

### 3.5. Independent Correlates of Energy Drink Consumption

A multivariable logistic regression model was developed to identify independent correlates of being an “ever” versus “never” consumer (Table 6). ROC analysis identified an optimal age cut-off of ≤53 years that best discriminated between ever and never energy drink consumers (AUC = 0.716, 95% CI 0.712–0.720) (Supplementary Figure S1).

In the final adjusted model, several factors remained strong, independent correlates of ever consuming an energy drink. The highest odds were associated with age ≤53 years (OR 3.80, 95% CI 3.61–4.01) and male sex (OR 3.17, 95% CI 3.03–3.32). Key behavioral factors also conferred strong association with consumption, including frequent fast-food consumption (OR 2.63, 95% CI 2.51–2.76) and being a current smoker (OR 2.49, 95% CI 2.23–2.77). Experiencing work exhaustion also remained a variable significantly associated with consumption, with a notable effect size (OR 1.64, 95% CI 1.56–1.72).

After adjusting for these covariates, the strong protective associations seen in univariate analyses for chronic conditions like hypertension (univariate OR 0.57) and heart disease (univariate OR 0.58) were substantially attenuated and lost practical significance (adjusted ORs of 0.88 for both), indicating that their initial effects were confounded mainly by age. Notably, the initial hypothesis that higher education would be associated with higher energy drink consumption was not supported in the final model.

## 4. Discussion

In this large-scale, nationwide study of 120,000 Polish adults, we provide a robust, high-resolution profile of energy drink consumption, revealing a behavior that, while not widespread in its most frequent forms, is firmly embedded within a specific demographic and lifestyle. Our principal finding is that energy drink use is not an isolated habit but a marker within a larger cluster of health risk behaviors, including smoking, poor diet, and problem drinking. This analysis, leveraging an extensive dataset, allowed us to dissect the independent correlates of consumption, confirming some established patterns while challenging others, and highlighting a strong dose-dependent relationship between consumption frequency and a wide array of adverse health symptoms.

The overall prevalence of ever having consumed an energy drink (16.9%) in our Polish sample aligns with the moderate range reported in global systematic reviews, which typically place general population prevalence between 15% and 35% [1,16]. Our findings powerfully reaffirm that the core consumer base consists of young and middle-aged men. The nearly four-fold increased odds for individuals aged 53 or younger and over three-fold increased odds for men are consistent across numerous international studies [1,17]. This demographic skew is likely a multifaceted phenomenon, driven by targeted marketing campaigns that associate energy drinks with masculinity, performance, and excitement, as well as lifestyle factors and occupational demands more prevalent among these groups [18]. A particularly noteworthy finding from our multivariate analysis was the lack of an independent association with education level. This contradicts a common hypothesis and suggests that the appeal and use of energy drinks are not confined by educational attainment, making a strong case for population-wide, rather than class-specific, public health messaging.

The central theme emerging from our data is the profound interconnection between energy drink use and other established health risk behaviors. The powerful, dose-dependent associations with current smoking (OR 2.49) and frequent fast-food intake (OR 2.63) were among the variables most strongly associated with consumption in our model. This suggests that energy drink consumption is deeply integrated into a broader lifestyle pattern that may be characterized by a lower prioritization of long-term health, a greater propensity for risk-taking, or both. This clustering is well-documented in the literature, where energy drink use has been linked to the consumption of other psychoactive substances and a range of risky behaviors [19]. The practice of mixing energy drinks with alcohol (AmED), for instance, is a major public health concern because the stimulant effects of caffeine can mask the subjective feeling of intoxication, leading to higher overall alcohol consumption and an increased risk of alcohol-related harm [20,21]. It should also be acknowledged that, given the relatively low prevalence of regular (weekly or more frequent) use in our data, a substantial proportion of harm at the population level may stem from intermittent, high-dose consumption in specific high-risk contexts (e.g., mixing energy drinks with alcohol, use during prolonged driving or intensive studying), as suggested by previous research, although these patterns could not be directly assessed in our dataset. The strong association with work exhaustion in our study adds another layer to this narrative, suggesting a functional motivation for use: individuals may be turning to energy drinks as a tool to cope with occupational stress and fatigue, potentially creating a maladaptive cycle of stimulant use to offset the consequences of poor sleep and burnout.

Our analysis further demonstrates a clear and concerning dose–response relationship between the frequency of energy drink consumption and the prevalence of adverse health symptoms. The strong, positive correlations with insomnia and daytime sleepiness are particularly compelling and biologically plausible. The high levels of caffeine in these products are known to disrupt sleep architecture, delay sleep onset, and reduce sleep quality, which in turn leads to daytime fatigue and a perceived need for further stimulant use to maintain functioning-a classic vicious cycle [22,23]. This sleep disruption is a likely contributor to the robust association we observed with mental health issues. The prevalence of depression rose from 17.1% in non-consumers to 36.5% in daily users, a finding that echoes a growing body of literature linking energy drink consumption to an increased risk of depression, anxiety, and psychological distress [24,25,26]. This relationship is likely multifactorial, stemming from the direct neurobiological effects of chronic high-dose caffeine intake, the indirect consequences of poor sleep on mood regulation, and the potential for shared underlying risk factors. However, given the cross-sectional design, these graded associations cannot establish the direction of effect. It is equally plausible that pre-existing sleep disturbances or depressive symptoms lead to higher energy drink use as a coping strategy, or that shared underlying factors (e.g., stress or adverse socioeconomic conditions) contribute to both.

Finally, the increased reporting of cardiovascular complaints, such as chest pain, among frequent consumers warrants careful consideration. Although our cross-sectional design cannot establish causality, the self-reported symptoms are consistent with the known physiological effects of energy drinks, as documented in controlled clinical trials and meta-analyses. These studies have shown that acute consumption can significantly increase systolic and diastolic blood pressure, elevate heart rate, and may contribute to endothelial dysfunction and cardiac arrhythmias [27,28]. Case reports have even linked excessive consumption to severe cardiovascular events, particularly in susceptible individuals [29]. Therefore, the symptoms reported by consumers in our study should be viewed as a potential real-world signal of these underlying physiological impacts, underscoring the clinical relevance of monitoring consumption patterns.

### Strengths and Limitations

This study possesses several key strengths that bolster the validity and importance of its findings. The most significant is the massive, nationwide sample size of 120,000 participants. This provides unparalleled statistical power, enabling the detection of subtle associations and the development of a robust multivariable model that can control for numerous potential confounders simultaneously. The national scope of the survey enhances the generalizability of our findings to the broader adult population of Poland, offering a far more comprehensive picture than smaller, regional studies. Furthermore, the survey collected data on a wide array of covariates, allowing for a detailed exploration of the sociodemographic, behavioral, and health-related context of energy drink consumption.

Despite these strengths, several important limitations must be carefully considered when interpreting the results. The most fundamental is the cross-sectional design of the study, which precludes any inference of causality. While we identified strong, dose-dependent associations between energy drink use and outcomes like depression and insomnia, we cannot determine the direction of these relationships. For instance, it is plausible that individuals with pre-existing sleep disturbances or depressive symptoms consume energy drinks as a form of self-medication to cope with fatigue, just as it is plausible that chronic consumption contributes to the onset or exacerbation of these conditions. Longitudinal, prospective studies are required to untangle these complex causal pathways.

Second, all data were collected via a voluntary, online self-report survey (CAWI), which introduces several potential sources of bias. Selection bias is a key concern; the sample, while large, is not a random probability sample of the Polish population. Individuals with internet access, higher digital literacy, and potentially a greater interest in health topics may be overrepresented, which could limit the generalizability of the prevalence estimates. Furthermore, self-reported data are susceptible to recall bias, as participants may not accurately remember the frequency of their consumption or symptoms, and social desirability bias, where respondents might under-report behaviors perceived as unhealthy, such as high-frequency energy drink use or problem drinking. The ‘ever use’ measure is based on self-report and may be affected by recall bias and social desirability, potentially leading to underestimation of true lifetime exposure.

Third, the measurement of the primary exposure-energy drink consumption-was limited in its granularity. The survey question captured the frequency of consumption but did not assess the quantity (e.g., volume consumed per occasion), the specific brands used, or the total daily intake of caffeine and other stimulants. This lack of detail prevents a precise dose–response analysis and makes it difficult to directly compare our findings with those of studies that employ more rigorous dietary assessment methods. Future research should aim to quantify consumption more precisely to better understand the threshold at which health risks increase.

Finally, while our multivariable model adjusted for a wide range of important covariates, the possibility of residual confounding from unmeasured variables remains. This includes, in particular, unmeasured socioeconomic factors, which may influence both energy drink consumption and related health outcomes. Factors such as personality traits (e.g., sensation-seeking, impulsivity), specific occupational demands, or underlying genetic predispositions could influence both the likelihood of consuming energy drinks and the health outcomes we examined. Additionally, some exposures available in the NHTP 2024 dataset, such as selected information on medication and dietary supplement use, were not included in the present analytic model to preserve its focus and interpretability; these factors may be explored in future dedicated analyses. Although our findings are robust after adjustment, these unmeasured factors may still account for some of the observed associations. In particular, unmeasured or not modeled factors such as physical activity or detailed socioeconomic gradients may contribute to residual confounding.

## 5. Conclusions

In conclusion, this large-scale national study provides compelling evidence that energy drink consumption in Poland is not a randomly distributed behavior but is concentrated within a well-defined, high-risk demographic of young to middle-aged men. Our most critical finding is that this consumption is deeply embedded within a cluster of adverse health behaviors, including smoking, poor diet, and work exhaustion. The strong, dose-dependent associations with a significant burden of negative outcomes—particularly sleep disturbances, poor mental health, and cardiovascular complaints—underscore the public health gravity of this consumption pattern.

These findings carry significant implications for public health policy and clinical practice. They suggest that interventions focused solely on the risks of energy drinks may be insufficient. Instead, a more holistic approach is required, one that addresses the complex lifestyle context in which consumption occurs. Public health strategies should therefore be designed to target this entire cluster of co-occurring risks, promoting healthier coping mechanisms for stress and fatigue and advocating for comprehensive lifestyle modification. This study provides a clear mandate for moving beyond single-issue messaging to address the multifaceted nature of this growing public health challenge.

### Implications

The findings of this study carry significant and actionable implications for public health policy, clinical practice, and the direction of future research. For public health and policy, our results strongly suggest that interventions focused solely on the risks of energy drinks may be insufficient. A more holistic approach is required, with campaigns designed to address the entire cluster of co-occurring risks, integrating messaging about smoking cessation, healthy nutrition, and stress management, particularly when targeting young men. This study provides a clear, population-level evidence base supporting regulatory actions, such as restrictions on marketing and the implementation of clear front-of-pack warning labels. Crucially, the lack of an association with education level implies that public health strategies must be broad and accessible, utilizing multiple channels to reach the entire population. Given the public health relevance, dissemination should extend beyond academic channels to public-facing media (e.g., social media, television, radio) to reach adolescents and young adults with clear, evidence-based messaging on co-use risks.

At the clinical level, these findings suggest that routine screening for energy drink consumption should be incorporated into standard lifestyle assessments, especially for young and middle-aged male patients. Frequent consumption can serve as a clinical “red flag,” prompting clinicians to screen for other underlying issues such as tobacco use, poor diet, burnout, depression, and sleep disorders. Healthcare providers are also in a key position to educate patients on the direct links between high caffeine intake and their symptoms, explaining how it can create a vicious cycle of poor sleep and daytime fatigue or exacerbate psychological distress.

Finally, this study highlights critical directions for future research. The most urgent need is for prospective, longitudinal studies to untangle the causal pathways between energy drink consumption and adverse health outcomes like depression and insomnia. Future investigations should also employ more granular methods to quantify consumption, including volume and total caffeine intake, to allow for precise dose–response analyses. Further research is also warranted to explore the physiological and neurobiological mechanisms underlying these associations and to employ qualitative methods to gain a deeper understanding of the motivations and social contexts driving consumption in high-risk groups.

## Figures and Tables

**Table 1:** Frequencies of responses to the question about drinking energy drinks and descriptive statistics values.

Q35. How Often Do You Drink Energy Drinks?	*n*	%
I never drink energy drinks (1)	99,750	83.13
Less than once a month (2)	11,893	9.91
Once a month (3)	2224	1.85
Several times a month (4)	2799	2.33
Once a week (5)	1058	0.88
Several times a week (6)	1530	1.28
1 to 2 times a day (7)	567	0.47
3 or more times a day (8)	179	0.15
M ± SD	1.34 ± 0.98	
Me [Q1; Q3]	1 [2]	
Min—Max	1–8	

M—mean; SD—standard deviation; Me—median (50%); Q1—lower quartile (25%); Q3—upper quartile (75%); Min—minimum; Max—maximum.

**Table 2:** Sociodemographic characteristics of respondents in groups differing in the frequency of energy drink consumption and Pearson’s Chi-squared goodness-of-fit test results.

Variable	Group				*p*-Value
	1 Never	2 Once a Month or Less	3 Once a Week or Less	4 At Least Once a Day	
Sex					<0.001
Women	66.4%	44.7%	43.6%	50.4%	
Men	33.6%	55.3%	56.4%	49.6%	
Age (year of age)					<0.001
18–24	0.9%	3.4%	10.3%	13.1%	
25–34	3.7%	12.5%	21.0%	23.2%	
35–44	10.5%	21.4%	24.9%	28.0%	
45–54	18.3%	24.5%	20.1%	18.8%	
55+	66.6%	38.1%	23.6%	16.9%	
Domicile					<0.001
Village	21.0%	20.1%	19.5%	17.6%	
City <20 k	12.9%	10.5%	9.5%	9.1%	
City <50 k	17.6%	13.9%	13.1%	15.4%	
City <100 k	12.3%	11.0%	10.2%	9.4%	
City <200 k	11.4%	10.5%	10.7%	11.4%	
City <500 k	10.7%	10.4%	10.9%	10.9%	
City ≥500 k	14.1%	23.6%	25.9%	26.3%	
Education					<0.001
Basic	11.3%	11.7%	13.8%	24.4%	
Secondary	41.4%	34.3%	39.3%	40.2%	
Higher	47.3%	54.0%	46.9%	35.4%	
BMI					<0.001
<18.5	1.9%	2.4%	3.6%	5.5%	
18.5–24.9	43.0%	42.2%	41.9%	41.7%	
25.0–29.9	30.8%	30.9%	28.0%	24.7%	
30+	24.2%	24.5%	26.4%	28.2%	
Current smoker	1.4%	5.3%	10.6%	16.2%	<0.001
Problem drinker	5.8%	9.9%	11.0%	16.0%	<0.001

**Table 3:** Characteristics of respondents in groups differing in the frequency of drinking energy drinks-prevalence of chronic diseases.

Health Problems	Group				*p*-Value
	1 Never	2 Once a Month or Less	3 Once a Week or Less	4 At Least Once a Day	
Chronic diseases	69.2%	58.5%	53.6%	60.2%	<0.001
Arterial hypertension	46.4%	35.4%	28.4%	26.9%	<0.001
Diabetes	14.2%	10.7%	9.9%	11.3%	<0.001
Heart disease	21.1%	14.3%	11.2%	13.8%	<0.001
COPD	4.7%	3.3%	3.0%	4.3%	<0.001
Allergy/asthma	25.0%	27.3%	27.9%	33.1%	<0.001
Depression	17.1%	21.6%	25.6%	36.5%	<0.001
Neoplastic disease	10.3%	6.5%	5.7%	4.4%	<0.001
Joint disease	32.5%	24.1%	20.4%	22.5%	<0.001
Neurological disease	17.7%	14.5%	13.4%	19.6%	<0.001
COVID-19	45.4%	48.1%	45.3%	39.7%	<0.001

**Table 4:** Characteristics of respondents in groups differing in the frequency of drinking energy drinks-prevalence of pain, sleep-related, and cardiometabolic symptoms.

Health Problems	Energy Drink Consumption Frequency				rho
	1 Never	2 Once a Month or Less	3 Once a Week or Less	4 At Least Once a Day	
Back pain					−0.069 ***
At least a few times	38.9%	38.6%	40.1%	50.1%	
Occasionally	43.5%	44.2%	42.0%	34.7%	
Never	17.6%	17.2%	17.9%	15.2%	
Headache					−0.022 ***
At least a few times	17.2%	21.6%	27.8%	34.9%	
Occasionally	55.7%	55.9%	52.0%	49.1%	
Never	27.1%	22.6%	20.2%	16.1%	
Chest pain					−0.019 ***
At least a few times	6.7%	7.1%	9.7%	13.7%	
Occasionally	34.4%	35.7%	34.0%	40.6%	
Never	59.0%	57.2%	56.3%	45.7%	
Heartburn					<0.001
At least a few times	13.2%	12.8%	13.6%	15.4%	
Occasionally	34.8%	38.4%	36.4%	40.2%	
Never	52.0%	48.8%	50.0%	44.4%	
Numbness of limbs					−0.002
At least a few times	23.9%	23.1%	25.4%	34.2%	
Occasionally	38.4%	38.8%	36.5%	34.0%	
Never	37.8%	38.0%	38.1%	31.8%	
Insomnia					0.032 ***
At least a few times	27.7%	23.7%	26.2%	36.2%	
Occasionally	40.0%	39.6%	37.5%	33.4%	
Never	32.3%	36.6%	36.2%	30.4%	
Daytime sleepiness					−0.086 ***
At least a few times	24.6%	31.3%	37.6%	46.8%	
Occasionally	42.5%	43.0%	39.5%	32.6%	
Never	32.9%	25.7%	22.9%	20.6%	
Excessive thirst					−0.057 ***
At least a few times	9.2%	11.9%	16.3%	25.7%	
Occasionally	31.5%	33.6%	33.1%	32.4%	
Never	59.2%	54.5%	50.6%	41.8%	
Blurred vision					0.001
At least a few times	18.4%	18.5%	19.8%	26.4%	
Occasionally	35.9%	35.9%	31.7%	29.4%	
Never	45.7%	45.6%	48.4%	44.2%	

***—*p* < 0.001.

**Table 5:** Characteristics of respondents in groups differing in the frequency of drinking energy drinks-prevalence of urinary, gastrointestinal, cognitive, and work-related symptoms.

Health Problems	Energy Drink Consumption Frequency				rho
	1 Never	2 Once a Month or Less	3 Once a Week or Less	4 At Least Once a Day	
Urinary incontinence					0.074 ***
At least a few times	12.5%	8.7%	7.8%	11.1%	
Occasionally	22.9%	18.8%	15.1%	18.0%	
Never	64.6%	72.5%	77.1%	70.9%	
Constipation					0.036 ***
At least a few times	14.8%	12.5%	12.7%	17.3%	
Occasionally	37.3%	34.8%	33.3%	38.9%	
Never	47.9%	52.8%	53.9%	43.8%	
Memory disorders					−0.003
At least a few times	11.9%	12.6%	14.4%	21.8%	
Occasionally	35.5%	34.9%	31.1%	28.7%	
Never	52.6%	52.5%	54.5%	49.5%	
Work exhaustion					−0.112 ***
At least a few times (3)	24.2%	32.8%	41.3%	54.4%	
Occasionally (2)	42.5%	43.0%	38.1%	27.9%	
Never (1)	33.3%	24.1%	20.6%	17.7%	

***—*p* < 0.001.

**Table 6:** Percentage of respondents in groups differing in energy drink consumption and analyzed risk factors, results of univariate and multivariate logistic regression analysis, and odds ratios with 95% confidence intervals.

Risk Factors	Energy Drink Consumption		Univariate Analysis		Multivariable Analysis	
	Ever	Never	b	OR [95% CI]	β	OR [95% CI]
Male sex	53.4%	33.6%	0.838	2.31 [2.22; 2.41]	1.155	3.17 [3.03; 3.32]
Age ≤ 53 years	64.5%	31.3%	1.384	3.99 [3.87; 4.12]	1.335	3.80 [3.61; 4.01]
City ≥ 500 k	24.3%	14.1%	0.673	1.96 [1.89; 2.03]	0.216	1.24 [1.18; 1.30]
Higher education	51.4%	47.3%	0.165	1.18 [1.14; 1.22]	-	-
BMI ≥ 32	16.2%	14.5%	0.126	1.13 [1.09; 1.18]	-	-
Current smoker	7.1%	1.4%	1.680	5.37 [4.98; 5.79]	0.911	2.49 [2.23; 2.77]
Problem drinker	10.4%	5.8%	0.642	1.90 [1.80; 2.00]	0.285	1.33 [1.23; 1.44]
Frequent fast-food consumption	40.0%	18.2%	1.100	3.01 [2.91; 3.10]	0.967	2.63 [2.51; 2.76]
Chronic diseases	57.2%	69.2%	−0.520	0.59 [0.58; 0.61]	−0.122	1.13 [1.08; 1.18]
Arterial hypertension	33.2%	46.44	−0.557	0.57 [0.55; 0.59]	−0.169	0.88 [0.85; 0.93]
Diabetes	10.5%	14.2%	0.334	0.71 [0.68; 0.74]	-	-
Heart disease	13.5%	21.1%	−0.539	0.58 [0.56; 0.61]	−0.126	0.88 [0.83; 0.94]
COPD	3.2%	4.7%	−0.396	0.67 [0.61; 0.73]	-	-
Allergy/asthma	27.6%	25.0%	0.139	1.15 [1.11; 1.19]	-	-
Depression	23.2%	17.1%	0.381	1.46 [1.41; 1.52]	-	-
Neoplastic disease	6.2%	10.3%	−0.559	0.57 [0.54; 0.61]	−0.209	0.81 [0.74; 0.89]
Joint disease	23.0%	32.5%	−0.478	0.62 [0.60; 0.64]	−0.114	0.89 [0.85; 0.94]
Neurological disease	14.4%	17.7%	−0.243	0.78 [0.75; 0.82]	-	-
COVID-19	47.0%	45.4%	0.065	1.07 [1.04; 1.10]	-	-
Ever back pain	82.7%	82.4%	0.001	1.02 [0.98; 1.06]	-	-
Ever headache	78.3%	72.9%	0.294	1.34 [1.29; 1.39]	-	-
Ever chest pain	43.5%	41.0%	0.099	1.10 [1.07; 1.14]	-	-
Ever heartburn	51.1%	48.0%	0.122	1.13 [1.10; 1.16]	-	-
Ever numbness of limbs	62.2%	62.2%	0.001	1.00 [0.97; 1.03]	-	-
Ever insomnia	63.7%	67.7%	−0.178	0.84 [0.81; 0.86]	-	-
Everdaytime sleepiness	75.2%	67.1%	0.398	1.49 [1.44; 1.54]	-	-
Ever excessive thirst	47.0%	40.8%	0.255	1.29 [1.25; 1.33]	-	-
Ever blurred vision	53.7%	54.3%	−0.026	0.97 [0.95; 1.01]	-	-
Ever urinary incontinence	26.3%	35.4%	−0.429	0.65 [0.63; 0.67]	−0.090	0.92 [0.87; 0.96]
Ever constipation	47.2%	52.1%	−0.193	0.82 [0.80; 0.85]	-	-
Ever memory disorders	47.1%	47.4%	−0.015	0.99 [0.96; 1.02]	-	-
Ever work exhaustion	77.0%	66.7%	0.516	1.68 [1.62; 1.74]	0.492	1.64 [1.56; 1.72]

b—univariate logistic regression coefficient, β—multivariate logistic regression coefficient, OR—odds ratio and its 95% confidence interval.

## Data Availability

The data used in this study originate from the archival database of the 2024 National Health Test of Poles (NTZP), obtained following approval from the appropriate Bioethics Committee. In accordance with NTZP regulations and the scope of participant consent, individual-level data (including anonymized records) are not publicly available and may not be shared with third parties.

## Supplementary Materials

The following supporting information can be downloaded at: https://www.mdpi.com/article/10.3390/nu17233747/s1, Figure S1. ROC curve for predicting the probability of ever consuming energy drinks based on respondents’ age, area under the curve (AUC), cutoff value, and sensitivity (Sn), specificity (Sp), and positive likelihood ratio (LR+).
