# Supplementary material for: A Cluster of Risks: Correlates of Energy Drink Consumption with Smoking, Diet, and Burnout in the Polish Adult Population
Source: Nutrients. 2025 Nov 28;17(23):3747. doi: 10.3390/nu17233747 (PMC12693943; doi:10.3390/nu17233747)

Supplementary Figure S1. ROC curve for predicting the probability of ever consuming energy drinks based on respondents' age, area under the curve (AUC), cutoff value, and sensitivity (Sn), specificity (Sp), and positive likelihood ratio (LR+).

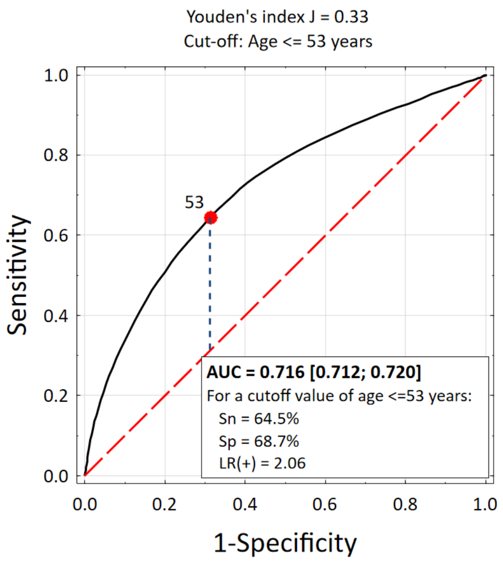

Supplement: Supplementary file 1 [file nutrients-17-03747-s001.zip › nutrients-3951272-supplementary.pdf]
